# Predisposed vulnerabilities and survival among the Finnish soldiers of World War II: historical life course approach

**DOI:** 10.3389/fsoc.2024.1495009

**Published:** 2025-01-03

**Authors:** Ville Kivimäki, Virva Liski, Ilari Taskinen

**Affiliations:** ^1^Research Department of the Finnish Literature Society, Helsinki, Finland; ^2^Faculty of Social Sciences, Finnish Research Council Centre of Excellence in the History of Experiences (HEX), Tampere University, Tampere, Finland; ^3^Tampere Institute for Advanced Study, Tampere University, Tampere, Finland

**Keywords:** Finland, history, life course approach, methodology, mortality, soldiers, war stress, World War II

## Abstract

In this methodological paper we propose a historical life course approach to analyze soldiers’ predispositions to experience war-related violence and stress and to respond to it. We argue that a closer quantitative inspection of pre-war and wartime factors will help to understand the various causes leading to different exposures to stress and violence during the war, which have consequently had different outcomes for the war survivors’ later lives. Our methodology is designed for a rich data source, the Finnish Army in World War II Database (FA2W, *N* = 4,253), but is generally also applicable to other case studies. We will demonstrate in practice how we apply the historical life course approach to the study of soldiers’ pre-war background variables, wartime service paths, and measurable war stress exposures. In the final discussion, as one potential follow-up to our proposal, we will point to an advanced historical analysis of community-building and meaning-making linked to different war experience profiles combining the quantitative social historical methodology with a qualitative cultural history approach.

## 1 Introduction

Research on war-related stress and trauma and their long-term impacts on the post-war mortality, health, and wellbeing of those affected is plentiful, but the results are mixed and partly contradictory. Follow-up studies on war survivors’ mortality, for example, have shown an elevated predisposition to cardiovascular disease in those groups of people who were subjected to severe war stress (Kunnas et al., 2011; Akosile et al., 2018; Al-Makhamreh et al., 2021). Mental health problems, permanent injury, heavy drinking, and smoking among war survivors have been recognized as predictors of late-life mortality (Bedard and Deschênes, 2006; Boscarino, 2006; Bramsen et al., 2007; Den Velde et al., 2011). Increased late-life mortality has been linked to some particularly stressful and traumatic war experiences, such as being a prisoner of war, having served at the frontlines in particularly stressful conditions, or experiencing war stress at a vulnerable age in adolescence (Horiuchi, 1983; Elder et al., 2009; Costa, 2012). Yet in contrast to these results, a large meta-analysis of war-related stress exposure and post-war mortality by Roelfs et al. (2010) found no increased population-level mortality risk associated with earlier war stress, and similar observations have been made concerning Finnish war veterans (Saarela and Finnäs, 2012; see also Leppik and Puur, 2020). Furthermore, in addition to the adverse or neutral effects of wars on the survivors’ post-war health and mortality, research on “post-traumatic growth” has argued for the positive outcomes of war experiences such as increased resilience, appreciation of life, and supportive social relations (Tedeschi and Calhoun, 1996). Personal strength and optimism associated with post-traumatic growth have been shown to correlate with such factors as gender, young age, good economic situation, and strong wartime unit cohesion in connection with exposure to combat (Kimhi et al., 2010; Mitchell et al., 2013; Sagi-Schwartz et al., 2013; cf. also Costa and Kahn, 2010).

The studies above suggest that while there may be a connection between war-related stress exposures and negative outcomes in later life, this link is not necessarily automatic and does not apply to all affected persons in the same way. There are various kinds of war stress – and different groups of people may experience the “same” stress differently. Furthermore, it seems evident that people have varying predispositions and resources to deal with their war experiences, and these factors, in turn, are connected to wider societal circumstances, social variables, and individual life courses.

In this methodological paper we want to take a step further and argue that in addition to various post-war factors affecting subjective coping with stress and trauma, the study of the long-term consequences of war would benefit from a closer look at those *pre-war* and *wartime* factors, which initially predispose people to different kinds of war stress, influence their experience of that stress and, moreover, construct various paths of survival and vulnerability in time of war. As far as we can see, these points have rarely been taken into account—most obviously due to a lack of sufficient data (for exceptions, see Fornasin et al., 2019; McCalman et al., 2019). We see the emphasis on pre-war and wartime factors as a way to gain a more profound and contextualized understanding of war stress, which is also crucial for interpreting its long-term outcomes.

Thus, we propose a historical multi-method approach to study the contextualized nature of war stress predisposition and experiences. In historical research, each method has to be modified to suit the particular case and the source materials available. While our own case study concerns the Finnish soldiers of World War II and this methodological proposal is designed particularly for a database representing the Finnish Army in World War II, our methodological approach is also applicable to other cases of war stress exposure if properly adjusted and if the sources allow. This methodological proposal is a research note. Due to ongoing data construction no preliminary results can be presented here.

In the history of military psychiatry, predispositions and vulnerability have varyingly meant constitutional and inherited qualities, membership of certain ethnic or socioeconomic groups, developmental circumstances in the childhood home and qualities related to war exposure, military leadership, unit cohesion, and military discipline (Shephard, 2004). The Vietnam War and the subsequent research emphasis on posttraumatic stress disorder (PTSD) brought the pre-selective role of draft policies to the center of predisposition claims leading to an emphasis on the role of social class as the main predisposing factor both in the sense that the lower classes were seen as carrying a disproportionate share of war burden – and in the sense that high rates of stress reactions would be attributable to soldiers’ class-related sociobiological vulnerabilities (Badillo and Curry, 1976; Appy, 1993; Dean, 1997; Shephard, 2004). Lately, vulnerability to war stress has been explained through biological markers varying between individuals (Yehuda and McFarlane, 1995; Bowman and Yehuda, 2004).

In our view predisposition and susceptibility to war stress have seldom been seriously studied from the perspective of wider sociopolitical factors and military policies, meaning that predisposition is often conceptualized as individual premorbid characteristics, which leads to different degrees of victim blaming disconnected from the issues of governance (on this claim in more recent conflicts, see Kriner and Shen, 2010; Kriner and Shen, 2016). Earlier conceptualizations of predisposition may be attributable in part to a lack of historical contextualization and suitable source materials, and partly also to political interests in presenting war as an equally demanding effort on the part of the whole nation (see Kriner and Shen, 2016).

The majority of research into the socioeconomic predispositions of military service and war deployment and their consequences on post-war life courses is conducted in the context of the US military. The US army, despite historical variations between draft and all-volunteer governance, is a system where voluntarism, subsequent enlistment, and selection play a vital role in the construction of the military personnel. This differs from all-male conscript armies in the era of world wars. For instance, in Finland in 1939–45, the vast majority of the male population was required to participate in military service. Since earlier studies have concentrated on pre-selection in the sense of who actually joins the army, less research has shed light on how men with different backgrounds and abilities were deployed *within* the system they had already entered in equal measure regardless of their socioeconomic status (for exceptions, see MacLean, 2011; MacLean, 2019).

The effect of the quality of war exposure on later-life outcomes has been studied in a multitude of settings, showing that combat veterans suffer worse health outcomes than non-combatant veterans and non-veterans (Schnurr and Spiro, 1999; Elder et al., 2009; MacLean, 2019). In military sociology, ending up as a combatant has been seen as a result of three alternative but often overlapping causes: class bias, which assumes that soldiers are exposed to combat because of their lower socioeconomic background; the human capital hypothesis, assuming that better educated and skilled people are spared from frontline service because their abilities are more useful elsewhere, e.g., in administration and communications; and lastly the impact of institutional screening, assuming that those at the bottom of the social ladder are actually at less risk of ending up in the military in the first place because armies conduct screening and selection, excluding men unsuitable for service (MacLean, 2011). These hypotheses have been studied in the US context concluding that those with lower parental socioeconomic status are at higher risk in facing combat regardless of enlistment selection, possibly because of their poorer ability to navigate inside the army system leading them to more dangerous tasks (MacLean, 2011). Those already at the bottom of society usually end up carrying the bulk of the combat burden, and the impacts of violence have been shown to vary according to their backgrounds and aptitudes. Occupational assignment has been shown to be linked with the risk of combat exposure and thus the risk of dying, being wounded, and being subjected to more intense war stress in the US army (Kriner and Shen, 2016, p. 568–569). Earlier research in Finland has also indicated that there were substantial regional differences in mortality and some also between social classes (Waris, 1948; Toivonen, 1998; Honkasalo, 2000).

History as a discipline has potential to combine fields of demography and (military) sociology by providing exceptional research datasets and detailed contextualization on the costs of war to individuals, communities, and even on entire nations. Historical demography has recently contributed to the questions above by providing and utilizing archive-based cradle-to-grave datasets on war generations. An Australian study using wide cohort data covering pre-war, wartime, and post-war socioeconomic and war service information found that better educated and taller Australian men were at greater risk of dying in World War I. They also found significant variation between service branches in probability of dying; men assigned to the infantry were at greatest risk. Importantly, the study observed that the previous life-course factors associated with risk of death usually persisted despite the war experience (McCalman et al., 2019). In their study on mortality differentials among Italian soldiers in World War I, Fornasin et al. (2019) used detailed individual level data covering socioeconomic, anthropometric, and war-deployment information, concluding that soldiers’ assignments to different tasks in war were dependent on socioeconomic status, where illiterate peasants were at the greatest risk of dying.

### 1.1 Historical background: Finland in World War II

**Figure 1:**
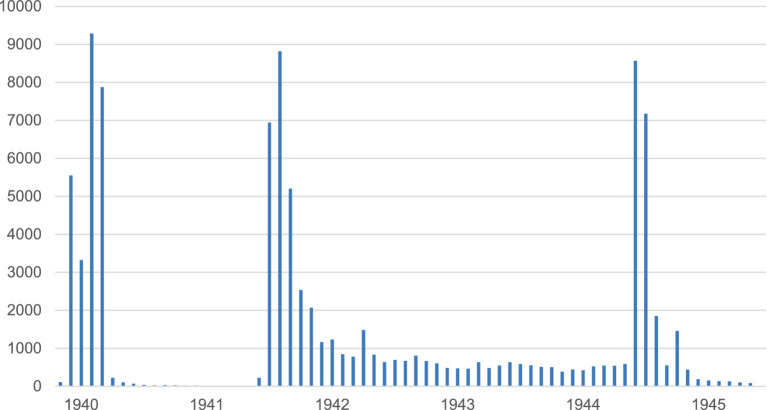
Monthly distribution of war deaths in the Finnish Army during World War II. Source: National Archives of Finland (2024).

In 1939–45, Finland waged three wars, each of them distinctive in character. First, the Winter War against the Soviet Union lasted from the end of November 1939 until mid-March 1940 and caused heavy casualties on both sides. After a peace of 15 months, the so-called Continuation War from June 1941 to September 1944 was characterized by two periods of intensive fighting against the Soviet army: first in the summer and autumn of 1941 and then in the summer of 1944, with a long and static war period in between. Third, in the Lapland War from September 1944 to April 1945, the Finnish Army fought against the retreating German troops in Northern Finland. While the initial stage of the war entailed full-scale combat, the overall death toll of the Lapland War was small compared to the two earlier conflicts. Figure 1 demonstrates how the Finnish war-related military deaths were distributed during World War II, with the three casualty peaks of the Winter War, the early Continuation War, and the summer of 1944 clearly visible:

All in all, about 96,000 Finns lost their lives due to war-related causes and over 200,000 were wounded, almost half of them permanently. These were staggering figures for a population of about 3.7 million and translates to a war mortality rate of 2.6%, which is roughly on the scale of the estimated global death toll caused by World War II (Honkasalo, 2000, p. 515; Kivimäki, 2018).

Yet the Finnish figures are also highly exceptional: about 98% of the war-related deaths were those of persons serving in the army. This is unique among any war-waging nation in Europe and equalled only in countries such as Australia, New Zealand, and the United States, which were, unlike Finland, located far away from the actual battlefields. About 2,000 Finnish civilians died in air raids, a small number of civilians were caught and interned by the Red Army in the early stages of the Winter War, while some civilian casualties were caused by Soviet partisan attacks on remote border villages during the Continuation War. Otherwise, the country’s civilian population at large did not have to face the consequences of occupation and being overrun by foreign armies. Finnish Jews were not murdered in the Holocaust. Furthermore, only a maximum of 4,500 Finnish soldiers became prisoners of war during the war (Frolov, 2004, pp. 11–12). While their mortality rate in Soviet captivity was very high, this remained a minor cause of death compared to the total death toll. The vast majority of war-related deaths in Finland in 1939–45 were directly linked to men’s military service at the front.^1^

These specific historical conditions provide some important advantages when studying war-related stress exposures. Compared to almost all other war-waging countries in 1939–45, the Finnish case allows one to focus on a relatively easily identified and controlled group of people, who experienced the heaviest war-related violence unlike other segments of society: the servicemen of the Finnish Army. Their war experiences obviously varied a lot depending on a multitude of factors; nevertheless, the general Finnish context of experiencing war-related violence was quite similar and consisted mostly of conventional frontline stressors such as combat, physical hardships, sleep deprivation, inadequate living conditions, psychological insecurity, and the long-term accumulation of all these variables. It would be too much to say that the quality of this war stress could be objectively standardized. Nevertheless, in the Finnish case the varieties of the heaviest war stress are relatively uniform if compared to countries, where World War II meant large-scale civilian suffering, ethnic and political persecution, massive POW experiences, deportations, and radical changes to the social and political systems.

Another important premise of the Finnish case is that soldiering touched men of all social classes and groups. As a small nation residing next to a totalitarian great power, Finland had to utilize her population to the absolute limits in military service, which meant universal male conscription from 1918 onwards. Every man was obligated to attend to the army call-ups at the age of 18–20. What is more, there exists a systematic collection of personal service records to study the Finnish soldiers’ war experiences, as will be described below. Such comprehensive materials are rarely if ever available for countries overrun and occupied by foreign armies and where administrative systems collapsed and archives were destroyed.

## 2 Materials

### 2.1 Finnish Army in World War II database (FA2W)

In 2023, the Research Council of Finland granted four-year funding for our research project “Unequal War: Vulnerability, Stress and Survival in the Finnish Army during World War II” (UnWar). The foundation of UnWar is the demographic “Finnish Army in World War II Database” (FA2W). This database consists of detailed personal, health, and military service information of c. 4,250 Finnish men who belonged to the 30 main birth cohorts (1897–1926) that the Finnish Army mobilized during World War II, with some additional older men who served or were inspected by the army. The database is representative of all Finnish men of these age groups, the Finnish war generation of World War II.^2^

The gathering of such an extensive database has been possible thanks to the military service record collection of the Finnish Army stored in the National Archives of Finland (NAF). The collection consists of personnel documents that the Finnish Army has produced on its men since the beginning of Finnish independence in 1917 and is extraordinarily representative. Due to strictly implemented universal conscription and the total wartime mobilization of Finnish society, practically all Finnish men of the above-mentioned cohorts were examined by the army and as many as 76% of them served during the war. The records have also survived very well: the collection holds files for 99% of Finnish men of the birth cohort 1903–26 who were alive at draft age, and around 70% of the birth cohorts 1897–1902. The missing men are mostly those who were exempt from military service or had died before World War II, thus records exist on practically every Finnish man who fought in the war.

The core of our database is based on a stratified random sample picked from the military service record collection. In the first phase of building the sample, we divided the population of the database (Finnish men of birth cohorts 1897–1926) into age groups based on the live number of men of draft age (as recorded in the vital statistics of Finland) with a sampling fraction of 250. Inside the birth cohorts men were further divided into those who were killed and those who survived the war. This division is possible because the National Archives of Finland has created a database of all Finnish military fatalities of World War II, which we discuss in the next section. The men were picked randomly from the index of the military service records and the database of the casualties.

To include background information on the men missing from the military service record collection (30% of the men of birth cohorts 1897–1902), we are in the process of taking an additional sample of these men from another source collection, the drafts records (*kutsuntaluettelo*) in which every Finnish man’s basic socioeconomic information was recorded for call-ups. When these two samples are combined, the database becomes representative of all Finnish men of the birth cohorts 1897–1926 who were alive at draft age (*N* = 4,253). It is our understanding that this is the most representative database of a male population of any nation taking part in World War II.

Descriptive statistics for the basic war exposure characteristics of the sample are presented in Table 1. Because the sample is drawn from the male population of draft age from 1919 onwards, some of the men categorized as having no war service had already died before the war. Some of the figures may still change because at the time of writing the data input is still ongoing for a small part of the sample.

**Table 1:** Finnish Army in World War II database: sample war exposure (*N* = 4,253).

	*n*	%
Died	377	8.93
Wounded	675	15.87
Survived unwounded	2,181	51.28
No war service	1,020	23.91
Total	4,253	100.00

The military service record collection comprises personnel files. A man’s file consists on average of 15 pages of service and medical records compiled during the call-up procedures, military training, wartime service, and postwar reservist period.^3^ The main document of the collection is a military service card (*kantakortti*) written for a man after a call-up and updated throughout his military career. The document contains the man’s personal information, military service history, evaluations, and details of war experience like being wounded and participation in battles. A man’s file typically contains numerous copies of this document, most of which were written during the war because updating the original card was difficult in the rapidly changing circumstances. Appendices include, for example, medical examination records, criminal files, and documents on treatment for illnesses and wounds.

The Finnish Army in World War II Database consists of over 60 different variables gathered primarily from the military service record collection. The variables include:

1. Socio-economic information from draft and wartime: date and place of birth, residence, occupation, education, marital status, number of children, mother tongue, religion, etc.
2. Longitudinal data on military career: service status, military unit, service branch, task, service class, and military rank.
3. Military training and war experiences: training, skills, evaluations, injuries, honors, punishments, participation in battle, death.
4. Medical information: health examinations, illnesses and ailments, medical treatments.

There are some limitations to the extent to which such information can be collected for men. Soldiers’ military career history is recorded in the documents with great precision, but details of their social and health backgrounds are at times incomplete. In general, the information has been recorded and archived meticulously for men entering service in the 1930s and 1940s but is more often missing for men conscripted in the early years of Finnish independence in the 1920s or exempted from service at call-ups. The omissions are the result of changing record-keeping and archival practices. For example, a medical examination record completed at call-up and updated throughout a man’s service, was only introduced in 1928.

The basic demographic characteristics of the sample collected so far are presented in Table 2. Data on occupation and education are taken from information recorded at a draft or at the beginning of conscription service. The gathering of this data is still ongoing, and we expect to have data on these variables for around 90% of the men once the data collection is completed.

**Table 2:** Finnish Army in World War II database: descriptive statistics of sample demographic characteristics (data input not yet completed).

	*n*	%/m/sd
Year of birth	3,989	1911.5 ± 8.96
Mother tongue		
Finnish	3,437	89.79
Swedish	383	10.01
Other*	8	0.21
Total	3,828	100.00
Place of birth (Province)		
Uusimaa	419	11.88
Turku and Pori	487	13.81
Häme	344	9.76
Viipuri	660	18.72
Mikkeli	232	6.58
Kuopio	473	13.41
Vaasa	489	13.87
Oulu	303	8.59
Lappi	116	3.29
Åland Islands	3	0.09
Total	3,526	100.00
Municipality type		
Urban	586	16.62
Market town	126	3.57
Rural	2,814	79.81
Total	3,526	100.00
Occupation (HISCLASS8)		
Students	163	5.51
Managers, professionals, clerical and sales	146	4.93
Foremen and skilled workers	336	11.36
Farmers and fishermen	848	28.66
Lower-skilled workers	206	6.96
Unskilled workers	150	5.07
Farm workers	460	15.55
General workers	650	21.97
Total	2,959	100.00
Education		
Higher education	154	5.13
Intermediate education	198	6.59
Elementary school	1,921	63.97
Little schooling	270	8.99
No formal education	460	15.32
Total	3,003	100.00

^*^The category “Other” includes single observations of language groups Sami, Russian, Estonian, and Turkish.

Regarding research ethics, all the information in the database is anonymized and the data is never represented in a form that would allow individual identification. We have been granted research permission from the National Archives of Finland, and all the researchers and research assistants in the project have signed a non-disclosure agreement and promised confidential treatment of the research data. Our data management plan has been approved by the responsible authorities of Tampere University, and we have conducted a data protection risk assessment preview. As almost all the war survivors in our sample, the youngest of them born in 1926, had died at the time of completing the database and as our research is based solely on archive data, no formal data protection impact assessment or ethical review have been required.

Our research team began to build the FA2W database during the research project “Large Databases in Studying the History of War Experiences” (STASKO), funded by the Finnish Cultural Foundation in 2017–20. With this funding, we took an initial sample for the database using a systematic random sampling method, photographed over 60,000 documents and inputted one quarter of the data with web-based database management platform REDCap (Harris et al., 2009; Harris et al., 2019).^4^ After the STASKO project, work on the database continued on a small-scale for 2 years, the greatest changes being the redefining of the sample to be a more accurately stratified random sample and some minor changes to the database. Now with the UnWar funding, the inputting of the data will be completed by the end of 2024.

In the future the FA2W database is envisioned to be enriched with new data from soldiers’ post-war lives. As a representative sample of the Finnish male population, the FA2W database also serves as a reference group for expansions of specific groups of people who are marginally represented on the scale of whole population. We are currently starting to collect the first additional set of men, a sample of 754 war casualties, that will triple the count of these men in the database. This sample is being taken to enable a more detailed analysis of war mortality among servicemen.

### 2.2 Other source materials

In addition to the military service record collection, we will utilize several other sources and datasets in our analyses. For the construction of the FA2W database, the second vital body of material is the draft records (*kutsuntaluettelot*). These records contain annual call-up lists from each Finnish municipality. They were compiled before call-ups in the military districts based on population register keepers’ information and updated with the men’s socioeconomic information, height and weight measurements, and call-up results on the call-up day. The draft records allow us to remedy the deficiencies of the military service record collection. We will use them to take an additional sample of men missing from the oldest birth cohorts and to gather socioeconomic information from call-ups for those men who are missing this data in the military service records.

Another important dataset is the database of war-related military deaths gathered by the National Archives of Finland. This public database which has been collected since the 1980s contains socioeconomic, military, and cause of death information for all approximately 94,000 Finnish military casualties of World War II (National Archives of Finland, 2024). The weakness of this impressively representative dataset is that there were previously no reference data on casualties for men who survived the war, and this was one of the prime reasons why we began to build the FA2W database. With our new database soon complete, the research prospects of the military deaths database will also improve in the analyses of the Finnish soldiers’ mortality.

Additionally, we will utilize archive material produced by the Finnish Army. As we are interested in demographic issues of war and military service, various personnel statistics on call-ups, conscription service, and the wartime army are important to us. There are also many questions relating to call-up decisions and allocations of human resources inside the army that need to be examined from different guidelines, orders, and reports for the background of our demographic analyses.

## 3 Methods: life course approach

Our anticipated analysis centers on the links between three types of data that record Finnish soldiers’ pre-war backgrounds, service paths, and war experiences. Our project falls under the scholarly tradition of *life course approach* (from now on LCA; Kok, 2007) that has become increasingly popular in historical demography in recent years. The LCA differs from the traditional demographic scholarship in that it focuses on the study of processes instead of mere landmarks of the human life course (e.g., birth, marriage, death). “Life course” can be defined as *standardized biography*, “a sequence of positions of a particular person in the course of time,” as demographic historian Kok (2007, p. 204) writes. LCA studies frequencies and timings of *events* or *transitions*, meaning changes in position in the course of human life. These transitions form *trajectories*, time series of altering roles and statuses. The approach is best suited for historical analysis since it also considers the interaction between individual lives and wider societal developments using the concepts of *social pathways* and *cultural scripts* to describe the normative patterns at certain times and places directing life courses.

The major advantage of LCA for our research setting is that in addition to studying correlates between subgroups and death, injury, and trauma we are able to study how men’s transitions from different roles and positions to others influenced their survival or exposure in war. The benefit is that instead of mere associations between background factors and outcomes, we are able to take into consideration time and transitions between them. In the forthcoming methods section, we present the three main steps of our forthcoming analysis, followed by a section of their anticipated results. In the discussion section, we suggest one possible way forward to combine social historical demographic research with cultural history perspectives.

### 3.1 Predisposed experiences: soldiers’ social and health backgrounds

LCA studies the ongoing impact of past experiences in ever altering contexts (Kok, 2007). Past experiences affect the way people behave and the ways their life courses progress. Tracing the effect that men’s pre-war socioeconomic statuses, health, and military careers had on their war experiences is central to our objectives. This entails tracking the social status and health of men when entering service. In our setting the war is not the starting point of exposure but an experience on which men embarked with differing socioeconomic backgrounds, health conditions, motivations, and cultural resources.

The FA2W database includes several categories of data on men’s backgrounds. The first group includes data on their social standing. The most basic variable of this group is men’s occupation, which is the base variable for social class classifications like HISCO, which we will utilize in our analysis (Leeuwen et al., 2002). Another related factor is education, which is particularly important for our case, since we are examining men of call-up age—18–20 years. At this point few men had entered occupations signaling high social standing, but in the Finnish pre-war and wartime context attaining high school or secondary education indicated that they were destined for the middle and upper classes of society. Other variables include places of birth and residence, mother tongue, religion, marital status, number of children, illegitimacy, and next of kin. We are collecting this data from two periods: the call-ups and wartime. Of these, the call-up information will be central because it presents a moment when all the men were in the same, comparable stage in their lives.

The FA2W database includes detailed information on men’s health. In the call-ups Finnish men were questioned, examined, and measured by a physician, and this information was recorded in the men’s medical examination card. In the questionnaire part men were asked about their and their family members’ medical history, e.g., if there had been tuberculosis or other illnesses in the family. Next, the physician checked the man’s physical condition from head to toe and recorded all abnormalities. Measurements included height, weight, and chest expansion. This data is unfortunately only available for some 55% of men because the medical examination card was not in use during the first 10 years of Finnish independence.

Lastly, we have a few types of data on men’s individual qualities and motivation for military service. Two of these—volunteering for service and membership of the voluntary militia, the Civil Guard (*suojeluskunta*)—indicate high motivation. As Finland practiced universal male conscription, every man had to participate in a call-up at ages from 18 and 20 years, but they had also the option to apply to join the army at the age of 17. Membership of the Civil Guard, which was the largest pre-war national civic organization with over 120,000 members at its peak, also indicated a right-wing or “nationalistic” political orientation due to its origin as the militia of the conservative classes who defeated the working-class revolutionary forces in the Finnish Civil War of 1918. Information on criminal charges and punishments, on the other hand, allows us to point out those men who likely did not share the ideals of patriotic military service or otherwise found it difficult to obey the rules of the army and society. The evaluations of conscript service in the military service record (marks for punctuality, diligence, perception, military development, and conduct) can also be used to divide men between these two spectra.

The statistical tools to be applied in studying associations between background factors and war experience outcomes are multivariate regression models. Since our data contain a multitude of variables possibly affecting the outcomes, this method will help us control for confounding factors and isolate the impact of specific variables.

### 3.2 Soldiers’ service paths

Central to our life course approach is forming a *service path* for each member of our sample. The service path consists of day-by-day monitoring of Finnish men’s service in the army. A man’s service path in the army consists of three core steps or periods: call-up, conscript service, and wartime service. The call-ups concerned every Finnish man. The outcome of a call-up was either acceptance for armed service or exemption, which might be defined as total, temporary, or for the duration of peacetime. Once a man began his conscript service, he joined a military unit, was assigned tasks, and acquired a military rank. During peacetime, men returned home about a year later as reservists after completing their conscript training. After this period, men could spend years with no contact with the army until they were called to arms at the outbreak of World War II in the autumn of 1939.

In the spring of 1940, after the short Winter War, most Finnish soldiers were demobilized for over a year. During the Continuation War of 1941–44, the men transitioned between military and civilian life, when the oldest age groups were demobilized and young ones began their service. Some birth cohorts were mobilized and demobilized altogether three times during the war years. War also brought military service to the lives of many previously exempted men. In order to survive a world war a small nation like Finland had to utilize all its human resources, which meant calling up all previously exempted men for re-examination during the war. Thousands of older men previously considered unfit for armed service ended up at war this way. The wartime field army was finally demobilized in the autumn of 1944, and the rest of the fighting against the last German troops in Northern Finland was conducted by young conscripts.

Thanks to the meticulous record-keeping practices of the army, we can follow Finnish men’s trajectories on this journey with great precision. The army recorded dated information on soldiers’ roles and transfers during their service in the military service records. We use this information to reconstruct their service paths through longitudinal data on six variables: position, military unit, service branch, task, military rank, and service class. “Status” is the main variable consisting of 16 fixed values reflecting a man’s status in the army and society. For example, before call-up a man’s status is “civilian,” in conscription service he becomes a “conscript,” and during wartime frontline service he belongs to the “field army.”^5^ Military unit, service branch, and rank are self-explanatory, while “task” is a soldier’s specific service role like “rifleman” or “platoon leader.” Service class is the health and fitness classification used by the army to evaluate men’s capabilities to perform in various military tasks. For all these variables, we collect dated changes from men’s call-up until at least the end of World War II.

Simply put, transitions in service paths start from the call-up time and end in a soldier’s death or final demobilization. The first transition is the allocation of men to those accepted for and those exempted from military service. The next transition is when a man was deployed to the front line or to service on the home front. Men ordered to serve on the front line were further assigned to less dangerous auxiliary duties or more dangerous combat duties. Those ending up participating in battle either died, were wounded, or survived, and lastly those who survived were either injured or uninjured. For each transition, there are a multitude of explanatory variables affecting the likelihood of transition.

It is known that an individual’s age at the time of transition affects the further life course. Exposure to violence in adolescence is known to cause elevated risk for later-onset psychological problems. On the other hand, a later stage in the individual and family time cycle (fatherhood, being married, fixed occupational and civilian identity) has been linked to increased rates of war-related stress reactions among older men (Kivimäki, 2013). Timing in relation to previous experiences is important in our setting. We presume that the war stress experience was very different for those men who first served in the Winter War, were called up again at the outbreak of the Continuation War and mobilized for the third time to the extremely harsh conditions in the summer of 1944, compared to young green recruits first seeing action in June 1944. Going back and forth between the safety of the family home and the adverse conditions at the front caused additional stress (Kivimäki, 2013). These interactions between individual time (age), family time (stage in the family cycle), and historical time (economic cycles, social change, variations in the intensity of war efforts) have to be accounted for in the study of war stress, but they also help to explain people’s varying responses to the experience of stress.

Following the service path structure and accounting for the whole Finnish Army (and basically all Finnish males in the birth cohorts 1903–1926), we can see the links between the risks of different transitions and character group memberships. We regard these paths as cumulative trajectories where one transition leads to the next, although we acknowledge transitions back and forth between positions, for instance, when those men previously exempted from military service were called up again for re-assessment. We analyze the predisposition compositions of each transition group and calculate the risk of each transition in the service path for each individual.

For hands-on methodology we shall apply the central statistical methodology of LCA*, event history analysis*, also known as survival analysis, for which Cox’s proportional hazards model is frequently used. This is a regression model that uses the length of time from one transition to the next as the dependent variable, the response being the occurrence of a discrete event in time. The risk of experiencing the event is predicted with explanatory variables (age, marital status, profession, education, literacy, domicile, military rank, etc.). Censors are the units that never experienced the event of interest. This model can also deal with covariates that alter their values during observation (such as military rank, marital status, and number of children), which makes it most suitable for analyzing happenings in historical time series.

### 3.3 Measuring war experiences and stress

Anecdotes from historical wars and systematic evidence from recent conflicts have shown that wars are detrimental to the mental health of a sizeable portion of soldiers, with some men developing PTSD that may haunt them for decades afterwards. Mental wounds of war are much more difficult to examine from the historical archive materials than deaths and physical injuries, which have been recorded in population registers and postwar medical reports. In most armies during the world wars, the experiences of combat were not widely recognized to cause mental damage and treatment for them was inadequate (e.g., Wessely, 2006). In Finland, for example, only around 2 % of the soldiers received treatment in military hospitals for psychiatric reasons during World War II (Ponteva, 1977; Kivimäki, 2013). This number is just the tip of the iceberg of the phenomenon and also a challenge for our study. In the future, our aim is to gather data specifically from the men who were treated for psychiatric causes in the military hospitals, but as our database is currently a representative sample of the Finnish male war generation, it also reflects the under-diagnosis of these injuries. By calculation, our database should include around 70 psychiatric casualties, which does not yet permit complex statistical analyses of the origins and nature of their experiences.

Although we have limited direct evidence of the soldiers’ traumas and mental injuries, the detailed service data of our database enables us to conduct meticulous calculations that offer indirect evidence for their emergence. Similar analysis has been conducted by Costa and Kahn (2010) in their study on the effects of war stress on longevity among veterans of the US Civil War. Costa and Kahn used the fraction of company killed by wounds, the number of company members killed, the number of those killed in a regiment and the maximum number of losses in a single battle as measures for war stress.

We do the same by examining soldiers’ exposure to the risk of violence, which offers indirect evidence of soldiers’ troubling mental experiences. Our calculations are primarily based on the *duration of exposure*, which can be scrutinized at several levels. In the most basic detail, we can look at the *number of days* that a man served in the army during the war. In the next stage, we can consider the branch of service. Different service branches correlated with different quantities and types of war stress: in the infantry, death and injury could be daily experiences, while service in coastal artillery or anti-aircraft troops could mean living a relatively uneventful life far away from daily dangers. Going further, we can inspect the varying exposures to combat within branches with the *task* variable. At this stage our sample starts to be divided into too small sections for most of the service branches, but this is a usable differentiation method at least among infantry, the largest branch. Sorting men between roles such as “rifleman,” “clerk,” or “horseman” enables us to distinguish heavy war stress survivors from auxiliary servicemen behind the immediate firing lines.

Calculating men’s duration of service in these roles and duties already offers us extremely detailed data to estimate the accumulation of burdening mental experiences. However, we can estimate them with even greater precision by taking into account the intensity of violence during men’s days in service. This is crucial in the Finnish case of World War II because the intensity of fighting fluctuated considerably between 1939 and 1945 (see Figure 1). The fighting was fierce in the winter of 1939–1940, in the summer and autumn of 1941, and in the summer of 1944, but for two and a half years between the beginning of 1942 and the summer of 1944 the Finnish-Soviet frontline was mostly quiet and casualties were a fraction of those in the intensive periods of the conflict.

We intend to measure the intensity of fighting through casualty rates. These will be calculated by comparing personnel strengths and casualty counts. The main dataset for this is the above-mentioned war death database gathered by the National Archives of Finland, which includes information on all Finnish military fatalities 1939–45. At the most general level, the casualty rate can be calculated for the whole army by comparing the changing strength of the Finnish Army (data available in army reports every 2 weeks) and daily casualty counts that can be counted from soldiers’ dates of death from the war death database. The same sources also enable calculations for service branches.

The war death database moreover allows us to calculate casualty rates at the level of a single unit because the dataset includes data on soldiers’ military units at the time of death. Here, calculations become somewhat less accurate because gathering the changing manpower for several thousands of Finnish military units from archival reports would be too laborious a task. However, fairly precise calculations can be made for basic military formations such as infantry regiments and battalions, which had a standardized and relatively stable strength. With this method it is possible to estimate casualty rates at unit level for at least the infantry soldiers. The basic assumption in this measure is that the unit casualty rates describe the intensity of combat and the imminence of the threat of death and injury for soldiers in that given unit. It may also be associated with the likelihood of witnessing the deaths and mutilation of fellow soldiers and friends, an experience known to be pivotal in the development of post-traumatic stress disorders (Tanielian and Jaycox, 2008).^6^

Wounding is known to be a major stressor in studies on the long-term impacts of war stress, also in the sense of later psychological hardship (Bramsen et al., 2007). Our data contain information about soldiers’ injuries, the dates and diagnoses of which were recorded in the military service records and treatment documents completed in field and military hospitals. We operationalize wounding first as a dichotomous variable (yes/no). The data, however, allow us to make a detailed diagnostic division for more precise analysis when necessary. Severe illnesses—reported in the same documents—also partly reflect the burden of violence. Furthermore, military service records include lists of battles in which a man had fought, yet these data suffer from heterogenous recording customs. A relatively simple quantitative method here is to count the number of battle mentions for each soldier.

When the army, military branch, and unit casualty rates are linked to soldiers’ service paths, we can estimate numerically approximately how much direct violence the men in our database had witnessed in the war. Earlier studies on war stress as an explanatory for later-onset outcomes such as PTSD and death consider war stress exogenic, meaning that the composition or characteristics of the military unit had no effect on their placement at the front and thus on how heavy losses each unit suffered (Costa and Kahn, 2010). By constructing summary measures for war stress, we intend to study what characteristics and unit compositions predisposed individuals and groups to undergoing more severe war experiences. From the perspective of risk, we can produce a measurement of their daily and cumulative chances of death and survival. With lacking medical reports on soldiers’ mental injuries, we cannot think of a more detailed way in which the harshness of their experiences can be studied. In the first stages of our project, we will use this data similarly to the data on death and look at how exposure to violence was distributed among different social groups. Another way to use this data is to study the impact of combat exposure on survival, e.g., if the chances of survival increased or decreased with the accumulation of experiences. In the future, when we gather data on soldiers’ post-war life histories, this data will become even more important as we can analyze its potentially detrimental effects on veterans’ long-term wellbeing such as mortality and social status.

## 4 Anticipated results: uneven distribution of violence and stress

It is necessary to underline that currently our data enables us to examine the impacts of war only to the end of World War II. It is the aim of our project to gather data on the men’s postwar life trajectories (e.g., their health, social status, family relations, and mortality) in the future, but at this point we are concentrating solely on the analysis of pre-war susceptibilities in relation to war experiences and different stress exposures.

We expect the results of UnWar to reveal a socially and individually uneven distribution of violence and stress in the Finnish Army. Our expectations are that differences in wartime mortality and exposure to stress were primarily the result of the inner workings of the army. Our hypothesis is that the Finnish Army operated as a social system allocating men from different backgrounds to different tasks and duties, which led to unequal experiences during the war, e.g., between men assigned to tasks and branches with high combat risk and men assigned to low-risk duties. In addition, we expect the inequalities to also have affected those who survived the war. One of the primary goals of our study is to show that the burdens of war beyond dying, indeed the accumulation of war stress, varied between different social groups. Nevertheless, we are also keen to see whether some pre-war vulnerabilities and social disadvantages could lead to *less* war stress, as in the case of conscripts exempted for health reasons and working-class soldiers despatched to vital duties in the war industry. It is also possible that factors such as membership of the Civil Guard, pointing to high motivation, conservative values, and stable social background, could correlate with higher exposure to war stress.

The expected results of the life course analysis are at this point more uncertain due to the novelty of our approach. We assume that there are certain patterns in the formations of service path trajectories based on the cumulative effect of socioeconomic status, health, military career, and war experience. There are hypotheses that issues such as recurring mobilizations and demobilizations and prolonged periods of service affected soldiers’ behavior and chances of survival in wars. These are questions that can only be answered once we complete our empirical analyses.

More inequalities are expected to emerge in the future when we move on to study the post-war consequences of war experiences. As noted in the introduction, studies on different wars and countries have yielded a wide range of long-term effects of war, and forecasting them in our Finnish context is at this point difficult. However, one of our strong hypotheses is that inequalities of war experiences have had lasting regional effects on Finnish society. As has been shown in earlier research on Finnish war mortality, the casualty rates were highest for those residing in the northern and eastern rural areas of Finland (Waris, 1948; Kivimäki, 2018), and these same regions faced difficult structural changes in post-war Finnish society, as witnessed, e.g., in unemployment rates, declining population, and higher mortality. The harsh war experiences may have contributed to these adversities.

Thus, in the long-term, the most intriguing avenue for the forthcoming research is to combine our historically rich and contextualized pre-war and wartime data with research on the post-war consequences of war stress. Once we have completed the analysis of the soldiers’ pre-war social backgrounds and the consequent distribution of war stress inside the Finnish Army in 1939–45, we shall have a solid basis for a follow-up study on their post-war lives. This would include the investigation of a multitude of variables related to war survivors’ wellbeing, social status, and health, as well as possible post-traumatic symptoms and behavioral problems. At that point it becomes crucial to consult war stress researchers in psychiatry, behavioral sciences, and neurobiology, with whom we plan to cooperate in the analysis of soldiers’ post-war life trajectories.^7^

## 5 Discussion: from demographic analysis to cultural meanings

As we study Finnish soldiers’ linked lives and experience profiles in relation to different service paths and susceptibilities during the war, our research will most likely recognize the emergence of various new groupings among the soldiers based, for instance, on the nature of their frontline service, military branch, injuries and battle experiences, or the duration of their service. In the advanced stage of the project, these findings will point to a cultural historical analysis of community-building. People have varying cultural means to make sense of their experiences and to give them meaning based on their socioeconomic status, age, gender, ethnicity, education, religion, home region, political ideology, and other identities. Studies on responses to trauma have suggested that the ability to experience violence and loss as meaningful and consistent with one’s identity, basic assumptions, and future expectations may protect survivors’ sense of self and support mental coping (Janoff-Bulman, 1992; Ehlers et al., 2000; Hautamäki and Coleman, 2001; Brewin, 2003) but that there is also great individual variation in how traumatic symptoms combine with negative or positive world assumptions (Bosscher et al., 2024). This runs parallel with the research on post-traumatic growth, which has shown that people’s abilities to gain strength from potentially traumatic experiences varies depending, for instance, on their age, gender, and economic situation (Kimhi et al., 2010; Mitchell et al., 2013). Meaning-making is not only a “soft” cultural factor, but in relation to potentially traumatic experiences it may have direct consequences for a person’s health, wellbeing, and arguably, even for mortality.

Once the statistical social historical analysis of our research data is complete, we plan to use cultural historical methodology to study how the emerging “experience groups” correlate with those cultural and ideological discourses employed in wartime Finland to imbue soldiers’ hardships with collective and private meaning. As a point of departure, we understand meaningful experiencing as a social process, where people form “communities of experience” by gaining and giving meanings to their similar experiences, thus constructing shared identities (cf. e.g. Koselleck, 1992; Buschmann and Reimann, 2001; Pahl and Kivimäki, 2024; for communities of experience as a research concept, see Kivimäki et al., 2023). There are ample research materials on Finnish wartime public and private discourses and how they shaped different meaning-making strategies for different groups of people. These include various commemorative and public speeches, sermons, propaganda materials, newspapers, so-called “frontline papers” produced and circulated among the troops, as well as private letters.^8^

The basic methodological guideline here will be to analyze which war experiences were collectively addressed and which were neglected; which social groups inside the army were highlighted and which remained on the margins; and what kind of meanings were attached to those stressful experiences and service paths that were recognized in the analysis of the FA2W database. Later on, the historical analysis of meaning-making and the consequent community building can be carried on to the post-war era: how the war experiences were collectively and locally addressed after the war and how the Finnish war veterans organized themselves both formally and informally around their past war experiences. Nevertheless, we shall only return to this theme at a later stage.

The forthcoming cultural historical analysis will underline the basic tenet of our research project: that the life events which lead to different vulnerabilities, the consequent experiences of war stress as well as the strategies for coping with them are all contextual, multifactorial phenomena. The historical life course approach can provide a useful method to understand their human and social complexity. It remains to be seen whether our research findings will emphasize the historically particular and contingent nature of experiencing war stress in Finland in 1939–45, or whether they will reveal similarities and parallels with other cases.

## Data Availability

The datasets presented in this article are not readily available because the Finnish Army in World War II database, which is the basis for this article, is currently under construction. Requests to access the datasets should be directed to ilari.taskinen@tuni.fi.

## Footnotes

^1^Not all military deaths were directly combat-related, though: about 12% of the casualties were categorized as not having been caused by “enemy action.” These fatalities could include servicemen’s illnesses, such as tuberculosis, or accidents, suicides, and intoxications (Kivimäki, 2018.)

^2^A detailed introduction to the construction of the FA2W database is Taskinen (2023).

^3^The average number of document pages in a man’s file in our initial sample was 14 (Taskinen, 2023, p. 56). Since then, we discovered that around a quarter of the men in our sample had in their files additional records that had not been delivered to us. These documents have now been collected as well and they raise the average number of pages to 15.

^4^For REDCap, see https://projectredcap.org.

^5^For the full-list of values (see Taskinen, 2023).

^6^There are also existing self-report questionnaire measurements for combat exposure, for example Combat Exposure Scale CES (Lund et al., 1984).

^7^As a parallel project to UnWar, we are already cooperating with epigenetics researchers in molecular biology to study the transgenerational effects of war stress. This is done by combining another sample of military service records with the population-based Cardiovascular Risk in Young Finns Study (LASERI), which includes extensive health and social data on three post-war generations of Finns. For more information, please see the website of TREES: Transgenerational Epigenetic Effects of War Stress, https://www.tuni.fi/en/research/trees-transgenerational-epigenetic-effects-war-stress.

^8^Practically all the wartime newspapers in Finland have been digitized and are available for research; in our earlier research project “Digital History and Handwritten Sources,” we also digitized a large collection of about 7,000 wartime letters in machine-readable form. It is thus possible to use new digital methods to study the correlations and differences between public speech and private meaning-making vis-à-vis the soldiers’ war experiences (see, e.g., Taskinen et al., 2022). We can also rely heavily on existing research literature on the cultural history of war, which has focused strongly on political, cultural, religious, ideological and private discourses in wartime Finland (e.g., Salminen, 1976; Kemppainen, 2006; Tepora, 2011; Pilke, 2012; Tilli, 2012; Kivimäki, 2013; Hagelstam, 2014; Taskinen, 2021).

## References

[ref1] Akosile W. Colquhoun D. Young R. Lawford B. Voisey J. (2018). The association between post-traumatic stress disorder and coronary artery disease: a meta-analysis. Australas. Psychiatry 26, 524–530. doi: 10.1177/1039856218789779, PMID: 30113869

[ref2] Al-Makhamreh H. Alkhulaifat D. Al-Ani A. Mafrachi B. Saadeh A. Al-Ani H. . (2021). The impact of war-related stress on coronary artery disease severity in war survivors: a SYNTAX study. Int. J. Environ. Res. Public Health 18:3233. doi: 10.3390/ijerph18063233, PMID: 33800972 PMC8004035

[ref3] Appy C. (1993). Working-class war: American combat soldiers and Vietnam. Chapel Hill: University of North Carolina Press.

[ref4] Badillo G. Curry G. D. (1976). The social incidence of Vietnam casualties: social class or race? Armed Forces Soc. 2, 5–32. doi: 10.1177/0095327X7600200305

[ref5] Bedard K. Deschênes O. (2006). The long-term impact of military service on health: evidence from world war II and Korean war veterans. Am. Econ. Rev. 96, 176–194. doi: 10.1257/000282806776157731, PMID: 29125728

[ref6] Boscarino J. A. (2006). Posttraumatic stress disorder and mortality among U.S. Army veterans 30 years after military service. Ann. Epidemiol. 16, 248–256. doi: 10.1016/j.annepidem.2005.03.009, PMID: 16099672

[ref7] Bosscher I. I. de la Rie S. M. van der Aa N. Boelen P. A. (2024). Profiles of posttraumatic stress disorder and negative world assumptions in treatment-seeking refugees. Eur. J. Psychotraumatol. 15:14915. doi: 10.1080/20008066.2024.2314915, PMID: 38353932 PMC10868437

[ref8] Bowman M. L. Yehuda R. (2004). “Risk factors and the adversity-stress model” in Posttraumatic stress disorder: Issues and controversies. ed. Rosen G. M. (Chichester, WS: Wiley), 15–38.

[ref9] Bramsen I. Deeg D. J. H. van der Ploeg E. Fransman S. (2007). Wartime stressors and mental health symptoms as predictors of late-life mortality in world war II survivors. J. Affect. Disord. 103, 121–129. doi: 10.1016/j.jad.2007.01.014, PMID: 17291593

[ref10] Brewin C. R. (2003). Posttraumatic stress disorder: Malady or myth? New Haven, CT: Yale University Press.

[ref11] Buschmann N. Reimann A. (2001). “Die Konstruktion historischer Erfahrung: Neue Wege zu einer Erfahrungsgeschichte des Krieges” in Die Erfahrung des Krieges: Erfahrungsgeschichtliche Perspektiven von der Französischen Revolution bis zum Zweiten Weltkrieg. eds. Buschmann N. Carl H. (Paderborn: Schöningh), 261–271.

[ref12] Costa D. L. (2012). Scarring and mortality selection among civil war POWs: a long-term mortality, morbidity, and socioeconomic follow-up. Demography 49, 1185–1206. doi: 10.1007/s13524-012-0125-9, PMID: 22968939 PMC3496009

[ref13] Costa D. L. Kahn M. E. (2010). Health, wartime stress, and unit cohesion: evidence from union Army veterans. Demography 47, 45–66. doi: 10.1353/dem.0.0095, PMID: 20355683 PMC3000013

[ref14] Dean E. T. (1997). Shook over hell: Post-traumatic stress, Vietnam and the civil war. Cambridge, MA: Harvard University Press. 10.1353/rah.1999.0019 11623715

[ref15] Den Velde W. O. Deeg D. J. H. Hovens J. E. van Duijn M. A. J. Aarts P. G. H. (2011). War stress and late-life mortality in world war II male civilian resistance veterans. Psychol. Rep. 108, 437–448. doi: 10.2466/02.10.16.PR0.108.2.437-448, PMID: 21675559

[ref16] Ehlers A. Maercker A. Boos A. (2000). Posttraumatic stress disorder following political imprisonment: the role of mental defeat, alienation, and perceived permanent change. J. Abnorm. Psychol. 109, 45–55. doi: 10.1037/0021-843X.109.1.45, PMID: 10740935

[ref17] Elder G. H. Clipp E. C. Brown J. S. Martin L. R. Friedman H. S. (2009). The lifelong mortality risks of world war II experiences. Res. Aging 31, 391–412. doi: 10.1177/0164027509333447, PMID: 20161074 PMC2743276

[ref18] Fornasin A. Breschi M. Manfredini M. (2019). Deaths and survivors in war: the Italian soldiers in WWI. Demogr. Res. 40, 599–626. doi: 10.4054/DemRes.2019.40.22, PMID: 16041089

[ref19] Frolov D. (2004). Sotavankina Neuvostoliitossa: Suomalaiset NKVD:n leireissä talvi- ja jatkosodan aikana. Bibliotheca Historica 91. Helsinki: SKS..

[ref20] Hagelstam S. (2014). Röster från kriget: En etnologisk studie av brevdialoger mellan frontsoldater och deras familjer 1941–1944. Åbo: Åbo Akademi University.

[ref21] Harris P. A. Taylor R. Minor B. L. Elliott V. Fernandez M. O’Neal L. . (2019). The REDCap consortium: building an international community of software platform partners. J. Biomed. Inform. 95:103208. doi: 10.1016/j.jbi.2019.103208, PMID: 31078660 PMC7254481

[ref22] Harris P. A. Taylor R. Thielke R. Payne J. Gonzalez N. Gonde J. G. (2009). Research electronic data capture (REDCap): A metadata-driven methodology and workflow process for providing translational research informatics support. J. Biomed. Inform. 42, 377–381. doi: 10.1016/j.jbi.2008.08.010, PMID: 18929686 PMC2700030

[ref23] Hautamäki A. Coleman P. G. (2001). Explanation for low prevalence of PTSD among older Finnish war veterans: social solidarity and continued significance given to wartime sufferings. Aging Ment. Health 5, 165–174. doi: 10.1080/13607860120038348, PMID: 11511064

[ref24] Honkasalo M. (2000). Suomalainen sotainvalidi. Helsinki: Otava.

[ref25] Horiuchi S. (1983). The long-term impact of war on mortality: old-age mortality of the first world war survivors in the Federal Republic of Germany. Population Bullet. UN 15, 80–92. 12265835

[ref26] Janoff-Bulman R. (1992). Shattered assumptions: Towards a new psychology of trauma. New York, NY: Free Press.

[ref27] Kemppainen I. (2006). Isänmaan uhrit: Sankarikuolema Suomessa toisen maailmansodan aikana. Bibliotheca Historica 102. Helsinki: SKS..

[ref28] Kimhi S. Eshel Y. Zysberg L. Hantman S. (2010). Postwar winners and losers in the long run: determinants of war related stress symptoms and posttraumatic growth. Community Ment. Health J. 46, 10–19. doi: 10.1007/s10597-009-9183-x, PMID: 19229610

[ref29] Kivimäki V. (2013). Battled nerves: Finnish soldiers’ war experience, trauma, and military psychiatry, 1941–44. Åbo: Åbo Akademi.

[ref30] Kivimäki V. (2018). “Raskaat numerot: Sodissa 1939–1945 kuolleet” in Suomen puolustusvoimat 100 vuotta. ed. Karjalainen M. (Helsinki: Edita), 378–383.

[ref31] Kivimäki V. Malinen A. Vuolanto V. (2023). Communities of experience. Digital Handbook of the History of Experience. New York, NY: Academic Press.

[ref32] Kok J. (2007). Principles and prospects of the life course paradigm. Annales Démographie Hist. 113, 203–230. doi: 10.3917/adh.113.0203, PMID: 18052372

[ref33] Koselleck R. (1992). “Der Einfluß der beiden Weltkriege auf das soziale Bewußtsein” in Der Krieg des kleinen Mannes: Eine Militärgeschichte von unten. ed. Wette W. (München: Piper), 324–343.

[ref34] Kriner D. L. Shen F. X. (2010). The casualty gap: The causes and consequences of American wartime inequalities. Oxford: Oxford University Press.

[ref35] Kriner D. L. Shen F. X. (2016). Invisible inequality: the two Americas of military sacrifice. Univ. Memphis Law Rev. 46, 545–635. https://ssrn.com/abstract=2823978

[ref36] Kunnas T. Solakivi T. Renko J. Kalela A. Nikkari S. T. (2011). Late-life coronary heart disease mortality of Finnish war veterans in the TAMRISK study, a 28-year follow-up. BMC Public Health 11:71. doi: 10.1186/1471-2458-11-71, PMID: 21284848 PMC3038159

[ref37] Leeuwen M. Maas I. Miles A. (2002). “HISCO” in Historical international standard classification of occupations (Leuven: Leuven University Press).

[ref38] Leppik L. Puur A. (2020). Longevity of world war II Estonian volunteers in the Finnish Army: A follow-up study of the impact of the post-war life course and repressions. Demogr. Res. 43, 1155–1184. doi: 10.4054/DemRes.2020.43.39, PMID: 16041089

[ref39] Lund M. Foy D. Sipprelle C. Strachan A. (1984). The combat exposure scale: A systematic assessment of trauma in the Vietnam war. J. Clin. Psychol. 40, 1323–1328. 6511942 10.1002/1097-4679(198411)40:6<1323::aid-jclp2270400607>3.0.co;2-i

[ref40] MacLean A. (2011). The stratification of military service and combat exposure, 1934–1994. Soc. Sci. Res. 40, 336–348. doi: 10.1016/j.ssresearch.2010.04.006, PMID: 21113325 PMC2990971

[ref41] MacLean A. (2019). Military service and the socioeconomic attainment of Frenchmen, 1940–1980. Res. Soc. Stratif. Mob. 61, 1–19. doi: 10.1016/j.rssm.2019.02.004

[ref42] McCalman J. Kippen R. McMeeken J. Hopper J. Reade M. (2019). Early results from the “diggers to veterans” longitudinal study of Australian men who served in the first world war: short- and long-term mortality of early enlisters. Historical Life Course Stu. 8, 52–72. doi: 10.51964/hlcs9307

[ref43] Mitchell M. M. Gallaway M. S. Millikan A. M. Bell M. R. (2013). Combat exposure, unit cohesion, and demographic characteristics of soldiers reporting posttraumatic growth. J. Loss Trauma 18, 383–395. doi: 10.1080/15325024.2013.768847

[ref44] National Archives of Finland (2024). Database on Finnish War Deaths 1939–1945. Available at: https://www.avoindata.fi/data/en_GB/dataset/suomen-sodissa-1939-1945-menehtyneet (Accessed July 26, 2024).

[ref45] Pahl K. M. Kivimäki V. (2024). Histories of emotions and experiences: studying soldiers’ letters, poems, and memoirs. Gesch. Ges. 49, 47–69. doi: 10.13109/gege.2023.49.1.47

[ref46] Pilke H. (2012). Korsu-uutisia! Rintamalehtien jatkosota. Helsinki: SKS.

[ref47] Ponteva M. (1977). Psykiatriset Sairaudet Suomen puolustusvoimissa vv. 1941–1944: Jatkosodan aikana sota- ja kenttäsairaaloissa hoidettujen sotilaspotilaiden epidemiologinen ja seurantatutkimus. Helsinki: University of Helsinki.

[ref48] Roelfs D. Shor E. Davidson K. Schwartz J. (2010). War-related stress exposure and mortality: a meta-analysis. Int. J. Epidemiol. 39, 1499–1509. doi: 10.1093/ije/dyq132, PMID: 20724455 PMC2992629

[ref49] Saarela J. Finnäs F. (2012). Long-term mortality of war cohorts: the case of Finland. Eur. J. Popul. 28, 1–15. doi: 10.1007/s10680-011-9246-x

[ref50] Sagi-Schwartz A. Bakermans-Kranenburg M. J. Linn S. van M. (2013). Against all odds: genocidal trauma is associated with longer life-expectancy of the survivors. PLoS One 8:e69179. doi: 10.1371/journal.pone.0069179, PMID: 23894427 PMC3722177

[ref51] Salminen E. (1976). Propaganda rintamajoukoissa 1941–1944: Suomen armeijan valistustoiminta ja mielialojen ohjaus jatkosodan aikana. Helsinki: Otava.

[ref52] Schnurr P. P. Spiro A. (1999). Combat exposure, posttraumatic stress disorder symptoms, and health behaviors as predictors of self-reported physical health in older veterans. J. Nerv. Ment. Dis. 187, 353–359. doi: 10.1097/00005053-199906000-00004, PMID: 10379722

[ref53] Shephard B. (2004). “Risk factors and PTSD. A Historian’s perspective” in Posttraumatic stress disorder: Issues and controversies. ed. Rosen G. M. (Chichester, WS: Wiley), 39–61.

[ref54] Tanielian T. Jaycox L. H. (2008). Invisible wounds of war: Psychological and cognitive injuries, their consequences, and services to assist recovery. Santa Monica, CA: RAND Corporation.

[ref55] Taskinen I. (2021). Social lives in letters: Finnish soldiers’ epistolary relationships, intimate practices, and emotionality in world war II. Tampere: Tampere University.

[ref56] Taskinen I. (2023). Construction of the Finnish Army in world war II database. Hist. Life Course Stu. 13, 44–60. doi: 10.51964/hlcs13565

[ref57] Taskinen I. Turunen R. Uusitalo L. Kivimäki V. (2022). Religion, patriotism and war experience in digitized wartime letters in Finland, 1939–44. J. Contemp. Hist. 57, 577–596. doi: 10.1177/00220094211066006

[ref58] Tedeschi R. G. Calhoun L. G. (1996). The posttraumatic growth inventory: measuring the positive legacy of trauma. J. Trauma. Stress. 9, 455–471. doi: 10.1002/jts.2490090305, PMID: 8827649

[ref59] Tepora T. (2011). Lippu, uhri, kansakunta: Ryhmäkokemukset ja -rajat Suomessa 1917–1945. Helsinki: University of Helsinki.

[ref60] Tilli J. (2012). The continuation war as a Metanoic moment: A Burkean Reading of Lutheran hierocratic rhetoric. Jyväskylä studies in education, psychology and social research 449. Jyväskylä: University of Jyväskylä.

[ref61] Toivonen T. (1998). War and equality: the social background of the victims of the Finnish winter war. J. Peace Res. 35, 471–482. doi: 10.1177/0022343398035004004

[ref62] Waris H. (1948). Suomalaisen yhteiskunnan rakenne. Helsinki: Otava.

[ref63] Wessely S. (2006). Twentieth-century theories on combat motivation and breakdown. J. Contemp. Hist. 41, 268–286. doi: 10.1177/0022009406062067, PMID: 39659294

[ref64] Yehuda R. McFarlane A. C. (1995). Conflict between current knowledge about posttraumatic stress disorder and its original conceptual basis. Am. J. Psychiatry 152, 1705–1713. doi: 10.1176/ajp.152.12.1705, PMID: 8526234

